# The relationship between health and mating success in humans

**DOI:** 10.1098/rsos.160603

**Published:** 2017-01-25

**Authors:** Yong Zhi Foo, Leigh W. Simmons, Gillian Rhodes

**Affiliations:** 1 ARC Centre of Excellence in Cognition and its Disorders, School of Psychology, University of Western Australia, 35 Stirling Highway, Crawley, 6009 Western Australia, Australia; 2 Centre for Evolutionary Biology and School of Animal Biology, University of Western Australia, 35 Stirling Highway, Crawley, 6009 Western Australia, Australia

**Keywords:** health, humans, mating success, sexual selection

## Abstract

Health has been claimed to play an important role in human sexual selection, especially in terms of mate choice. Our preferences for attractive individuals are said to represent evolved adaptations for finding high-quality, healthy mates. If this is true, then we expect health to predict mating success in humans. We tested this hypothesis using several important physiological indicators of health, including immune function, oxidative stress and semen quality, and self-reported measures of sexual behaviour that contribute to mating success. In contrast to our hypothesis, we did not find a relationship between the physiological measures of health and sexual behaviour. Our results provide little support for claims that health, at least the health measures we used, increases mating success in relatively healthy humans.

## 1. Introduction

Health has been proposed to play an important role in sexual selection in humans [1–7]. In terms of mate choice, it has been suggested that our preferences for attractive individuals evolved as adaptations for finding high-quality mates. One important aspect of quality is health [1–11]. Healthy mates can provide direct benefits, including better parenting and more resources, are less likely to harbour parasites or pathogens, and are more likely to be fertile [8–9]. Healthy mates can also provide indirect genetic benefits to offspring, namely genes that code for good health [8,10–11]. Health can also influence sexual selection via mating competition [12–14]. Healthy individuals are able to invest more time and effort into competing with their same-sex conspecifics over access to the opposite sex. They may also have more resources for developing physical traits that enhance competitive success, such as physical size and dominance. If health is under sexual selection, then we expect healthier individuals to enjoy greater mating success than less healthy individuals.

The idea that health plays a role in sexual selection, particularly female mate choice, has been studied extensively in non-human animals. Several hypotheses have been proposed linking different physiological indicators of health to female mate choice. One of the earliest hypotheses was the Hamilton–Zuk hypothesis [11], which proposed that females select males with elaborate sexual signals because those signals provide reliable information about the males' ability to resist parasites. More recently, it was suggested that male sexual signals may also signal levels of oxidative stress in males [15,16]. Oxidative stress refers to the extent of cellular damage caused by excessive production of reactive oxygen species during respiratory processes [15,16]. Oxidative stress has been argued to play a role in sexual selection because of its involvement in various aspects of health [15,16] with links to diseases such as cancer, as well as to the shortening of telomeres, which are markers in the DNA of cellular lifespan [17,18]. Oxidative stress also negatively impacts immune function and sperm quality by damaging immune and sperm cells [16,19]. Finally, the phenotype-linked fertility hypothesis proposes that male signals provide information about semen quality [20,21], which is an indicator of male reproductive health and fertility, as well as more general health [22,23].

There is considerable evidence that sexual signals indicate immunity or parasitic infection in a variety of species, including birds, mammals, fishes, reptiles and insects (for reviews, see [24–29]). Furthermore, a meta-analysis revealed that carotenoid-colour-based sexual signals were positively correlated with immune function in birds [30]. Many animal studies have also investigated the relationship between male sexual signals and ejaculate quality, although a recent meta-analysis suggested that the overall relationship across species may be small and non-significant [31].

Although much of the focus in the animal literature has been on mate choice, health is also associated with mating competition [12]. For example, parasitic infection is negatively associated with mate competition behaviours, such as dominance, competitive social interactions, territory defence and stamina in several species [32–35].

Together, the animal studies suggest that health, or at least some aspects of health, may be subject to sexual selection via both mate choice and mating competition. However, for health to be subject to sexual selection, its impact on mate choice and mating competition has to translate into mating success. Therefore, we expect a positive relationship between health and mating success (however, see [36,37] for models showing that it is possible to find null or even negative relationships between health and mating success under indirect models of sexual selection). There is indeed supporting evidence from a number of taxa, including insects, birds and mammals showing that physiological indicators of health are positively associated with mating success [38–41]. For example, in drumming wolf spiders, *Hygrolycosa rubrofasciata*, males that were successful at mating have higher immune lytic activities compared to males that were not [40], while in rhesus macaques, *Macaca mulatta*, males with stronger innate immune responses and lower oxidative damage had higher mating success [41].

In humans, health has been linked to sexual selection via mate choice for facial attractiveness. Three attractive traits have been proposed to account for this relationship: sexual dimorphism, averageness and symmetry. There are good theoretical reasons to think that these traits may be honest signals of health. Sexual dimorphism is proposed to signal health due to trade-offs with health through the effects of testosterone [42]. Testosterone has been found to compromise immune functioning (see [43] for a recent meta-analytic review) and elevate oxidative stress [44]. Therefore, it has been argued that sexual dimorphism signals health because only healthy individuals can afford the high levels of testosterone needed for signal development. Testosterone is also linked to semen quality because it is essential for sperm production [45]. Averageness and symmetry are argued to indicate underlying genetic quality, and the ability of individuals to undergo normal development when subject to environmental stresses [1,46,47].

Various studies have examined the relationship between health and facial appearance in humans using a range of measures associated with health. Early studies linked facial appearance to perceived health [48], self-reported health conditions (e.g. number of infection/flu episodes and antibiotic use) [49,50] and health ratings by doctors based on assessments of the individual's medical records [48]. More recently, studies have examined the relationship between facial appearance and physiological indicators of health, such as immune function and oxidative stress. Antibody production in response to a hepatitis-B vaccination was found to be positively related to facial attractiveness and masculinity in men [51]. Indicators of oxidative damage to DNA and lipids were negatively related to facial attractiveness, symmetry and masculinity in men [52], and semen quality was found to be positively related to facial attractiveness [53] and masculinity [54] in men.

Relationships between facial appearance and variables associated with health support the argument that health may be sexually selected via mate choice in humans. Moreover, facial appearance is associated with self-reported measures of sexual behaviours that contribute to mating success (e.g. self-reported age at first sexual intercourse and number of sexual partners) (e.g. [55–57]). These measures have been related to attractive facial traits, such as sexual dimorphism, averageness and symmetry [55–57], consistent with sexual selection acting on putative signals of health.

Despite the evidence linking health to attractiveness and attractive traits to mating success, it remains unclear whether health is associated with mating success. So far, only one study has investigated the relationship between health and mating success in humans [58]. It measured health using genes at the major histocompatibility complex (MHC), which regulates adaptive immunity [58]. MHC genes code for molecules that bind to pathogens to present them to immune cells to be killed and for antibodies to be created. Greater MHC diversity, which may indicate a greater ability of the adaptive immune system to detect and kill a greater variety of pathogens, was related to the number of sexual partners in women [58]. No studies have investigated the relationship between health and mating success using physiological measures of immune function. Nor has there been any examination of whether mating success is related to physiological measures associated with health, such as oxidative stress and semen quality.

The main aim of this study was to investigate whether health is related to sexual behaviours that contribute to mating success in humans using physiological indicators of health that have not been studied previously. First, we measure innate immune function using salivary lysozyme level and salivary bacteria-killing capacity. Second, we measure oxidative stress using urinary 8-OHdG and 8-isoprostane levels, which are markers of DNA and lipid oxidative damage, respectively. Third, we measured semen quality. The measures used have been linked to various health outcomes, suggesting that they are valid indicators of health. For example, lysozyme level is associated with reduced gingivitis [59] and reduced susceptibility to acute bronchitis [60]. Increased 8-OHdG levels are associated with conditions such as cancer, inflammation, atherosclerosis and diabetes [61]. Likewise, increased isoprostane levels are associated with a range of disorders, including atherosclerosis, diabetes, asthma and lung disease [62]. Semen quality is associated with the history of fever episodes [63], mortality [64] and circulatory disorders [65], and of course affects male fertility [23].

A positive relationship between physiological indicators of health and age at first sexual intercourse or number of sexual partners would indicate that health may indeed be subjected to sexual selection in humans. We also examine the relationship between facial attractiveness and sexual behaviours to replicate previous findings that attractiveness is associated with mating success.

One could argue that self-reported measures of sexual behaviour may not accurately reflect reproductive success (e.g. the number of offspring). Measuring reproductive success using the number of offspring is subject to a number of problems. First, it is difficult to quantify without longitudinal data. Second, there is potential uncertainty of paternity without genetic testing. Lastly, sexual behaviour may not translate into reproductive success in modern Western societies where contraceptive usage is widespread. Nevertheless, we assume that our measures provide proxies for reproductive success and note that a number of studies have found positive relationships between attractive appearance and actual reproductive success [66,67], validating our measures as proxies for reproductive success.

## 2. Material and methods

### 2.1. Participants

One hundred and one male (mean age ± s.d. *=* 20.8 ± 3.6 years, range = 18–35 years) and 80 female (mean age ± s.d. = 21.9 ± 4.6 years, range 18–35 years) Caucasians were recruited from the University of Western Australia community in exchange for either course credit or transport remuneration (AUD10 + extra AUD5 for male participants who delivered their semen samples).

### 2.2. Procedure

Full details of the methods can be found in [54], from which the current sample was taken. Briefly, participants first attended a 1 h testing session. Participants provided a sample of saliva for innate immune function measurements. A sample of urine was provided for oxidative stress measurements. A photograph of each participant's face adopting a neutral expression with his/her mouth closed was taken. The faces were rated by a separate group of opposite-sex raters on their attractiveness using a 9-point scale (1 = Not attractive at all, 9 = Extremely attractive). Participants also completed a sexual history questionnaire, which included questions on their age at first sexual intercourse and their lifetime number of sexual partners. Hereafter we refer to these variables as ‘mating success’. Participants also completed a lifestyle questionnaire, which was used to identify potential lifestyle confounds that might impact the physiological measures. Male participants provided a sample of their semen on a separate day after the first testing session.

### 2.3. Salivary immune function

We measured salivary immune function using two assays: antibacterial capacity against *Escherichia coli* and lysozyme activity against *Micrococcus lysodekticus*. The antibacterial capacity assay consisted of three variables, namely bacteria-killing capacity, bacteria growth suppression capacity and overall antibacterial capacity. The four immune function variables were reduced to two factors for both sexes using principal components analyses (PCAs; see the electronic supplementary material for details). For men, PC1 was weighted most strongly by bacterial-killing capacity and overall bacterial immunity, and PC2 was weighted most strongly by bacterial suppression capacity and lysozyme activity. For women, PC1 was weighted most strongly by bacterial-killing capacity, overall bacterial immunity and lysozyme activity, and PC2 was weighted most strongly by bacterial suppression capacity.

### 2.4. Oxidative stress

We quantified the urinary 8-OHdG and 8-isoprostane levels using enzyme-linked immunosorbent assay kits (Northwest Life Science Specialties, Vancouver, WA, USA). The results were standardized against creatinine (i.e. urine) concentration in terms of ng mg^−1^ creatinine.

### 2.5. Semen quality

Semen quality was measured using the Hamilton Thorne computer-aided sperm analysis system. The system measures sperm concentration, percentage motile sperm and seven motility-related variables that were reduced into three factors using PCA (see the electronic supplementary material for details). PC1 was weighted most strongly by variables related to rapid progressive motility. PC2 was weighted most strongly by variables related to the linearity of the sperm movement. PC3 was weighted most strongly by sperm concentration and percentage motile sperm.

## 3. Results

The means and s.d.s of the attractiveness, mating success and physiological health indicator variables are presented in table 1. After running the PCAs to reduce the physiological data, General Linear Models were conducted on each of the physiological predictors for each sex separately to identify and control for potential lifestyle and sample collection confounds (see the electronic supplementary material for details). Age at first sexual intercourse in males and the number of sexual partners in both sexes were markedly non-normal. Therefore, we removed all outliers that were three standard deviations from the mean in each variable and ran all inferential statistics by bootstrapping based on 1000 samples and report bias-corrected and accelerated 95% confidence intervals (CIs) [68].

**Table 1.** Descriptive statistics, including means and s.d.s of the attractiveness, mating success and physiological variables. VAP, average path velocity; VSL, straight line velocity; VCL, velocity along the sperm cells point-to-point track; ALH, lateral amplitude of sperm head movement; BCF, frequency with which the sperm head crosses the average sperm path; STR, straightness of the sperm's path; LIN, linearity of the sperm's path.

	men		women	
	mean *±* s.d.	*N*	mean *±* s.d.	*N*
attractiveness	3.3 *±* 0.9	101	3.5 *±* 0.8	79
mating success				
age at first sexual intercourse	17.2 ± 2.5	86	16.9 ± 1.7	67
number of sex partners	5.6 ± 8.4	101	5.5 ± 7.0	77
immune function				
lysozyme activity	0.51 ± 0.11	98	0.51 ± 0.11	78
bacterial killing capacity	7.95 ± 37.78	98	0.48 ± 41.78	78
bacterial suppression capacity	20.84 ± 16.37	98	21.96 ± 25.47	78
overall bacterial immunity	29.66 ± 23.64	98	18.28 ± 15.88	78
oxidative stress				
8-OHdG	6.45 ± 3.23	98	7.67 ± 3.70	79
8-isoprostane	2.28 ± 1.60	101	1.91 ± 1.37	80
semen quality				
VAP	46.93 ± 8.98	91		
VSL	37.61 ± 7.79	91		
VCL	69.90 ± 13.51	91		
ALH	5.32 ± 1.06	91		
BCF	13.03 ± 1.86	91		
STR	77.78 ± 9.44	91		
LIN	54.87 ± 8.92	91		
concentration	89.72 ± 68.38	91		
% motile sperm	45.20 ± 17.50	91		

Zero-order correlations between the variables in this study are presented in table 2. A small number of physiological predictors were significantly related. In men, immune factor 1 was positively related to 8-isoprostane levels, immune factor 2 was negatively related to semen factor 3 and semen factor 1 was negatively related to semen factor 2 (table 2). In women, immune factor 1 is negatively related to 8-isoprostane levels and immune factor 2 is negatively related to 8-OHdG levels (table 2). Age was significantly correlated with age at first sexual intercourse and number of partners in men and number of partners in women (table 2). Therefore, all subsequent analyses were conducted while controlling for age.

**Table 2.** Zero-order Pearson product–moment correlations, *p*-values and *N*s between age, mating success, attractiveness and physiological indicators of health. Male results are below the diagonal. Female results are above the diagonal.

	age	age at first sexual intercourse	number of sex partners	attractiveness	immune PC 1	immune PC 2	8-OHdG	8-isoprostane	semen PC 1	semen PC 2	semen PC 3
age	—	0.20	0.54	−0.27	0.05	−0.15	−0.02	0.14	—	—	—
		0.10	0.00	0.01	0.64	0.20	0.86	0.22			
		67	75	79	77	78	78	79			
age at first sexual intercourse	0.44	—	−0.27	0.02	0.17	0.00	−0.06	0.10	—	—	—
	0.00		0.03	0.88	0.18	0.99	0.64	0.43			
	83		63	67	65	66	66	66			
number of sex partners	0.31	−0.28	—	−0.14	−0.05	−0.10	0.10	0.15	—	—	—
	0.00	0.01		0.24	0.70	0.40	0.39	0.19			
	97	84		74	72	73	73	74			
attractiveness	−0.07	−0.19	0.23	—	−0.36	0.12	−0.19	0.20			
	0.52	0.09	0.02		0.002	0.32	0.09	0.07			
	97	85	100		76	77	77	78			
immune PC 1	0.08	0.12	−0.07	−0.16	—	−0.07	0.06	−0.31	—	—	—
	0.46	0.28	0.49	0.11		0.53	0.58	0.01			
	94	83	97	98		77	76	76			
immune PC 2	−0.16	0.00	−0.10	0.04	0.00	—	−0.38	0.16	—	—	—
	0.13	0.99	0.35	0.72	1.00		0.00	0.17			
	94	83	97	98	98		76	77			
8-OHdG	−0.08	0.02	−0.07	0.20	−0.02	−0.10	—	−0.12	—	—	—
	0.43	0.85	0.47	0.05	0.84	0.32		0.30			
	95	82	98	98	95	95		77			
8-isoprostane	0.36	0.05	0.10	0.11	0.32	−0.17	−0.01	—	—	—	—
	0.00	0.66	0.32	0.30	0.00	0.11	0.93				
	95	83	98	99	96	96	97				
semen PC 1	−0.17	−0.07	−0.05	0.01	0.20	−0.03	−0.01	−0.10	—	—	—
	0.11	0.54	0.63	0.95	0.07	0.79	0.96	0.35			
	84	76	86	87	84	84	84	85			
semen PC 2	0.12	0.00	0.08	0.18	−0.04	0.17	0.06	0.12	−0.30	—	—
	0.26	0.98	0.46	0.09	0.70	0.12	0.60	0.26	0.00		
	82	74	84	85	83	83	82	83	85		
semen PC 3	0.22	−0.03	0.21	0.01	0.03	−0.23	−0.14	0.12	0.09	0.01	—
	0.04	0.81	0.05	0.95	0.81	0.03	0.20	0.27	0.43	0.91	
	88	78	90	91	88	88	88	89	87	85	

We first ran multiple regressions to examine whether attractiveness predicted mating success after controlling for age. We then ran multiple regressions to examine the relative contributions of the health variables to mating success after controlling for age. As some of the physiological predictors were related to each other, we calculated the variance inflation factor (VIF) for each physiological predictor in the multiple regression models to check whether our multiple regression models were subjected to the problem of multicollinearity. All the VIFs were less than 2, which were substantially lower than the recommended maximum of 10 [69]. Therefore, it was unlikely that our multiple regression models suffered from issues of multicollinearity.

### 3.1. Attractiveness and mating success

Multiple regressions results indicated that, in men, attractiveness positively predicted the number of sexual partners but not age at first sexual intercourse after controlling for age (number of sexual partners: B(s.e.) = 1.75(0.63), *t*_94_ = 2.77, *p* = 0.007, 95% CI [0.49,3.01]; age at first sexual intercourse: B(s.e.) = −0.29(0.22), *t*_80_ = −1.29, *p* = 0.20, 95% CI [−0.68,0.05].) In women, attractiveness did not significantly predict either number of sex partners or age at first sexual intercourse after controlling for age (number of sexual partners: B(s.e.) = 0.09(0.55), *t*_71_ = 0.16, *p* = 0.87, 95% CI [−0.92,1.14]; age at first sexual intercourse: B(s.e.) = −0.003(0.25), *t*_64_ = −0.01, *p* = 0.99, 95% CI [−0.53,0.44]).

### 3.2. Physiological indicators of health and mating success

Multiple regression results indicated that neither age at first sexual intercourse nor number of partners was significantly predicted by any of the physiological indicators in either men (table 3) or women (table 4).

**Table 3.** Multiple regression models for the physiological indicator predictors of male mating success. Results were based on bootstrapping with 1000 samples and bias-corrected and accelerated 95% CIs.

	B ± s.e.	*p*	95% CI for B	*t*(d.f.)	*r*	95% CI
age at first sexual intercourse						
age	0.36 ± 0.09	0.01	[0.10,0.60]	4.03(59)	0.46	[0.25,0.63]
immune factor 1	0.24 ± 0.24	0.33	[−0.25,0.84]	1.00(59)	0.13	[−0.11,0.36]
immune factor 2	0.01 ± 0.25	0.96	[−0.42,0.43]	0.04(59)	0.01	[−0.23,0.25]
8-OHdG	0.07 ± 0.09	0.37	[−0.07,0.23]	0.81(59)	0.10	[−0.14,0.33]
8-isoprostane	−0.38 ± 0.21	0.16	[−0.88,0.25]	−1.82(59)	−0.23	[−0.44,0.01]
semen factor 1	−0.23 ± 0.26	0.34	[−0.71,0.17]	−0.88(59)	−0.11	[−0.34,0.13]
semen factor 2	−0.10 ± 0.29	0.78	[−0.73,0.55]	−0.33(59)	−0.04	[−0.28,0.20]
semen factor 3	−0.10 ± 0.24	0.72	[−0.61,0.47]	−0.41(59)	−0.05	[−0.29,0.19]
number of sex partners						
age	0.62 ± 0.26	0.10	[0.04,1.36]	2.40(68)	0.28	[0.06,0.47]
immune factor 1	−0.63 ± 0.68	0.22	[−1.77,0.36]	−0.92(68)	−0.11	[−0.33,0.12]
immune factor 2	−0.07 ± 0.62	0.91	[−1.02,0.81]	−0.11(68)	−0.01	[−0.23,0.21]
8-OHdG	−0.16 ± 0.21	0.36	[−0.50,0.10]	−0.76(68)	−0.09	[−0.31,0.14]
8-isoprostane	0.42 ± 0.60	0.46	[−0.62,1.58]	0.71(68)	0.09	[−0.14,0.31]
semen factor 1	0.17 ± 0.70	0.81	[−1.16,1.38]	0.24(68)	0.03	[−0.20,0.25]
semen factor 2	0.44 ± 0.73	0.61	[−1.06,2.77]	0.60(68)	0.07	[−0.16,0.29]
semen factor 3	0.37 ± 0.67	0.57	[−0.82,1.46]	0.55(68)	0.07	[−0.16,0.29]

**Table 4.** Multiple regression models for the physiological indicator predictors of female mating success. Results were based on bootstrapping with 1000 samples and bias-corrected and accelerated 95% CIs.

	B ± s.e.	*p*	95% CI for B	*t*(d.f.)	*r*	95% CI
age at first sexual intercourse						
age	0.07 ± 0.05	0.15	[−0.03,0.15]	1.42(58)	0.18	[−0.07,0.41]
immune factor 1	0.35 ± 0.23	0.12	[−0.05,0.87]	1.52(58)	0.20	[−0.05,0.42]
immune factor 2	0.00 ± 0.24	1.00	[−0.45,0.54]	−0.01(58)	0.00	[−0.25,0.25]
8-OHdG	−0.03 ± 0.23	0.87	[−0.47,0.39]	−0.14(58)	−0.02	[−0.26,0.23]
8-isoprostane	0.18 ± 0.20	0.33	[−0.14,0.60]	0.89(58)	0.12	[−0.13,0.36]
number of sex partners						
age	0.60 ± 0.13	0.00	[0.37,0.92]	4.63(64)	0.50	[0.30,0.66]
immune factor 1	−0.10 ± 0.56	0.85	[−1.20,1.25]	−0.18(64)	−0.02	[−0.25,0.22]
immune factor 2	−0.07 ± 0.58	0.92	[−1.21,0.96]	−0.11(64)	−0.01	[−0.24,0.23]
8-OHdG	0.64 ± 0.61	0.26	[−0.46,1.68]	1.06(64)	0.13	[−0.11,0.35]
8-isoprostane	0.29 ± 0.54	0.69	[−0.81,1.83]	0.54(64)	0.07	[−0.17,0.30]

## 4. Discussion

We found no evidence to support the hypothesis that health is related to mating success. Health has been suggested to influence human mate choice through its influence on facial attractiveness [44–52]. We replicated previous findings that facial attractiveness was positively related to the number of sexual partners in men [56]. For health to be subject to sexual selection via mate choice for attractive individuals, we should also find positive relationships between health and mating success. We examined this relationship using three potential physiological indicators of health that were theoretically linked to sexual signals. We used multiple measures for each physiological indicator, which should provide a more robust measure compared to single measures [70,71]. In contrast to predictions, we did not find a relationship between physiological indicators of health and mating success. Thus, we found no evidence that health is sexually selected in the human population under study.

Our results run counter to those of a previous study, which found MHC diversity, which is associated with immune functioning, to be positively related to the number of sexual partners in women [58]. One distinction between the two studies is that the present study used indicators of current health, whereas MHC diversity in [58] represents a predisposition to long-term health. Maintaining health or invoking a physiological response to health threats (e.g. parasites or oxidative stress) is costly. Therefore, unlike genetic predisposition to health, current health is subjected to trade-offs with other life-history traits, such as reproduction [72]. Depending on the marginal fitness benefit of diverting resources from current health to reproduction, high genetic quality individuals might invest so much resources in reproduction that they end up being no healthier than lower genetic quality individuals. It is possible that such trade-offs might explain why we did not observe a relationship between our physiological indicators of health and mating success.

It might, however, be premature to conclude that current health is unrelated to mating success. It is possible that other indicators of health not assessed here may have been related to mating success. The immune system is a complex system made up of multiple physiological processes, including innate immune function, which provides the first line of defence against pathogens with its rapid and non-pathogen-specific responses, and adaptive immune function, which provides protection against future infections by the same pathogen by generating pathogen-specific responses. In the present study, we measured innate immune function and not adaptive immunity. However, it is possible that adaptive immunity could be linked to mating success. Indeed adaptive immunity (measured using antibody production in response to a hepatitis-B vaccination) has been linked to facial attractiveness and masculinity in men [51]. MHC diversity in genes associated with adaptive immunity in women has also been related to the number of sexual partners [58]. Therefore, a potential fruitful research direction will be to examine whether adaptive immunity predicts mating success in humans.

It is also possible that health is linked to mating success in other populations. Our participants were sampled from an Australian university community, which has good access to high-quality food, shelter and modern medicine. Preferences for facial signals of good health presumably evolved during times when environmental conditions were tougher and health was generally poorer. Therefore, it is possible that health and our preferences for signals of health might have been sexually selected in the past, but are no longer under current selection in modern, healthy populations. Indeed, health and preference for signals of health might still be sexually selected in populations where poor health has a greater impact on survival (e.g. environments with high prevalence of parasites) [73,74]. Human preference for male facial masculinity is stronger in countries with a lower National Health Index score ([73]; but see [75] for opposite findings), suggesting that there is greater selection pressure for signals of health in countries with poorer health. Therefore, another fruitful direction would be to compare the relationship between health and mating success across different countries to examine whether health has a stronger influence on mating success in countries with poorer health.

Our results offer no support for the view that our preferences for attractive individuals are adaptations for seeking healthy mates, which has been highly influential in evolutionary accounts of facial attractiveness in humans [1–7]. Recently, there has been an increase in attention to the possibility that our facial preferences might have arisen via intra-sexual selection (i.e. male-to-male or female-to-female competition) [13,14]. Puts [14] argued that our relatively large body size, the two-dimensionality of our environment, and comparative evidence that our great ape cousins experience intense intra-sexual competition are all in line with the proposal that humans, especially males, are subjected to strong intra-sexual selection pressures. Therefore, it has been suggested that instead of signalling health, male facial appearance might signal intra-sexual competitiveness [13]. Men with more masculine faces have been found to look more dominant [76], to react with stronger testosterone responses during a competition task [77], and to exhibit more aggression both in a laboratory and in a naturalistic setting involving players in university and professional hockey [78]. Therefore, men with more masculine faces might be more attractive because they are better at competing with other males for mates through increased dominance or aggressiveness, rather than because they are healthy.

One potential limitation of our study is that each of our physiological indicators of health was only measured once. Single measures might be susceptible to temporal fluctuations, which might add noise to the data and limit our ability to detect significant relationships between the physiological indicators and sexual behaviour. However, a previous study has shown that at least for urinary oxidative stress markers, single measures taken during laboratory testing produce results that are comparable to composite measures that were averaged across multiple measurements [52]. Nevertheless, it would be informative for future studies to replicate this study using composite measures of the physiological indicators.

In summary, we found no evidence to support the idea that health is related to mating success. Our results provide no support for the influential idea that health is sexually selected in humans. However, we cannot rule out the possibility that other measures of health may be related to mating success in other populations. To further understand the relationship, future research will need to investigate other measures of health and to study populations that vary more in health.

## Supplementary Material

The relationship between health and mating success in humans Electronic Supplementary Material - PCA results

## Data accessibility

The dataset for this study is available at https://researchdataonline.research.uwa.edu.au/handle/123456789/3301.
