# Supplementary material for: The relationship between health and mating success in humans
Source: R Soc Open Sci. 2017 Jan 25;4(1):160603. doi: 10.1098/rsos.160603 (PMC5319324; doi:10.1098/rsos.160603)
Supplement: The relationship between health and mating success in humans Electronic Supplementary Material - PCA results [file rsos160603supp1.docx]

**The relationship between health and mating success in humans**

Yong Zhi Foo^1,2,*^, Leigh W. Simmons^1,2^, and Gillian Rhodes^1^

^1^*ARC Centre of Excellence in Cognition and its Disorders, School of Psychology, University of Western Australia, 35 Stirling Hwy, Crawley, 6009, WA, Australia*

^2^*Centre for Evolutionary Biology & School of Animal Biology, University of Western Australia, 35 Stirling Hwy, Crawley, 6009, WA, Australia*

Electronic supplementary material

The following information has been reproduced from [1], from which the physiological data was taken.

1. Data reduction

Principal components analysis (PCA) was used to summarize the inter-related semen quality data and immune function data. Separate PCAs were conducted for male and female immune function data. Both sexes’ data returned two PCs with eigenvalues > 1 (Table S1). For men, PC1 was loaded mainly by bacterial killing capacity and overall bacterial immunity and PC2 was loaded mainly by bacterial suppression capacity and lysozyme activity. The two PCs jointly explained 80.15% of the variation in immune function variables. For women, PC1 was loaded mainly by bacterial killing capacity, overall bacterial immunity and lysozyme activity and PC2 was loaded mainly by bacterial suppression capacity. The two PCs jointly explained 85.03% of the variation in the variables.

Table S1. Means, *SD*s and factor loadings for immune function principal components analyses.

|  | Men | | | |  | Women | | | |
| --- | --- | --- | --- | --- | --- | --- | --- | --- | --- |
|  | M±SD | N | PC1 | PC2 |  | M±SD | N | PC1 | PC2 |
| Eigenvalue |  |  | 1.92 | 1.29 |  |  |  | 2.17 | 1.24 |
| % variance explained |  |  | 47.92 | 32.24 |  |  |  | 54.14 | 30.89 |
| Lysozyme activity | 0.51±0.11 | 98 | 0.34 | 0.63 |  | 0.51±0.11 | 78 | 0.73 | 0.41 |
| Bacterial killing capacity | 7.95±37.78 | 98 | 0.96 | −0.24 |  | 0.48±41.78 | 78 | 0.80 | −0.58 |
| Bacterial suppression capacity | 20.84±16.37 | 98 | −0.24 | 0.87 |  | 21.96±25.47 | 78 | −0.03 | 0.93 |
| Overall bacterial immunity | 29.66±23.64 | 98 | 0.90 | 0.28 |  | 18.28±15.88 | 78 | 0.93 | −0.15 |

The semen quality PCA returned three PCs with eigenvalues > 1 (Table S2). The three PCs jointly explained 88.28% of variation in the semen quality variables. PC1 was weighted most strongly by variables related to rapid progressive motility. PC2 was weighted most strongly by variables related to the linearity of the sperm movement. PC3 was weighted most strongly by sperm concentration and percentage motile sperm.

Table S2. Descriptive statistics and factor loadings for semen quality principal components analysis. VAP = average path velocity, VSL = straight line velocity, VCL = velocity along the sperm cells point-to-point track, ALH = lateral amplitude of sperm head movement, BCF = frequency with which the sperm head crosses the average sperm path, STR = straightness of the sperm's path, and LIN = linearity of the sperm's path.

|  | *M*±*SD* | *N* | PC1 | PC2 | PC3 |
| --- | --- | --- | --- | --- | --- |
| Eigenvalue |  |  | 4.91 | 1.82 | 1.22 |
| % variance explained |  |  | 54.53 | 20.25 | 13.50 |
| VAP | 46.93±8.98 | 91 | 0.76 | 0.43 | 0.41 |
| VSL | 37.61±7.79 | 91 | 0.62 | 0.57 | 0.40 |
| VCL | 69.90±13.51 | 91 | 0.95 | 0.19 | 0.21 |
| ALH | 5.32±1.06 | 91 | 0.91 | 0.03 | −0.02 |
| BCF | 13.03±1.86 | 91 | 0.35 | 0.84 | −0.10 |
| STR | 77.78±9.44 | 91 | 0.22 | 0.94 | 0.03 |
| LIN | 54.87±8.92 | 91 | −0.07 | 0.96 | 0.20 |
| Concentration | 89.72±68.38 | 91 | 0.00 | 0.01 | 0.90 |
| % motile sperm | 45.20±17.50 | 91 | 0.46 | 0.11 | 0.76 |

2. Data quality control for physiological predictors

We ran General Linear Models (GLM) on each of the physiological predictors for each sex separately to identify and control for variables that might confound our health and mating success results. We entered lifestyle variables from the lifestyle questionnaire as predictors (e.g. long-term or current medical condition, medicine usage, alcohol consumption, exercise frequency etc.). For the semen quality PCs, we also entered collection procedure variables (proportion ejaculate collected, time taken to collect, time elapsed between collection and analysis, and time since last ejaculation) and sample abnormalities (liquifaction before analysis and abnormalities under the microscope such as clumping) as predictors (see Table S3 for details). We employed stepwise deletion to identify the best model and the significant predictors.

For the male data, significant models were found for semen PCs 1 and 2 (PC1: *F*_3,79_ = 8.32, *p* < 0.001; PC2: *F*_5,77_ = 6.48, *p* < 0.001). PC1 was significantly predicted by the proportion of ejaculate collected (*β* = 0.28, *t*_79_ = 2.82, *p* = 0.006), time taken to collect the ejaculate (*β* = 0.31, *t*_79_ = 3.11, *p* = 0.003), and time since last ejaculation (*β* = −0.26, *t*_79_ = −2.64, *p* = 0.01). PC2 was predicted significantly by exposure to hot conditions (*β* = 0.22, *t*_77_ = 2.18, *p* = 0.03) and time since last ejaculation (*β* = −0.43, *t*_77_ = −3.95, *p* < 0.001) and non-significantly by current medication intake (*β* = 0.19, *t*_77_ = 1.95, *p* = 0.06), time elapsed between ejaculation to analysis (*β* = −0.20, *t*_77_ = −1.94, *p* = 0.06), and the frequency of sexual activity (*β* = −0.20, *t*_77_ = −1.85, *p* = 0.07). Residuals for these two health variables were extracted controlling for the variables that were retained in each of their stepwise deletion final model.

For the female data, significant models were found for 8-OHdG levels (*F*_3,72_ = 7.40, *p* < 0.001) and immune function PC1 (*F*_2,73_ = 5.45, *p* = 0.006). 8-OHdG level was predicted significantly by zinc intake (*β* = −0.26, *t*_72_ = −2.47, *p* = 0.02) and hours seated everyday (*β* = −0.38, *t*_72_ = −3.69, *p* < 0.001) and non-significantly by long term health condition (*β* = 0.19, *t*_72_ = 1.87, *p* = 0.07). Immune function PC1 was predicted significantly by anaemia (*β* = −0.37, *t*_72_ = −3.23, *p* = 0.002) and non-significantly by long term medical condition (*β* = 0.22, *t*_72_ = 1.93, *p* = 0.06). Residuals for these two health variables were extracted controlling for the variables that were retained in each of their stepwise-deletion final models.

Table S3. Details of lifestyle, collection procedure (semen quality only), and sample variables (semen quality only) used as control variables for actual health variables.

| Item | Response |
| --- | --- |
| Alcohol intake | 5-point scale |
| Antibiotics intake | Yes/No |
| Caffeine intake | 5-point scale |
| Current medication intake | Yes/No |
| Exercise frequency | 4-point scale |
| Hours seated per day | 6-point scale |
| Long term medical condition | Yes/No |
| Recreational drugs intake | Yes/No |
| Anaemia* | Yes/No |
| Hormonal contraceptive usage* | Yes/No |
| Zinc intake* | Yes/No |
| Regular exposure to hot conditions† | Yes/No |
| Sexual activity frequency† | 5-point scale |
| Stable romantic relationship† | Yes/No |
| Wearing tight clothing† | Yes/No |
| Observed abnormalities in semen sample† | Yes/No |
| Amount of ejaculate collected† | % of total ejaculate |
| Ejaculate liquified before analysis† | Yes/No |
| Time taken from ejaculation to analysis† | minutes |
| Time since last ejaculation† | hours |
| Time taken to collect ejaculate† | minutes |

*Used in female health analysis only because there was no variation among males.

†Variables specific to male semen quality analysis.

**References**

1. Foo YZ, Simmons LW, Rhodes G. in press Predictors of facial attractiveness and health in humans. *Sci. Rep.*
